# An in-depth analysis of the personal factors and their pathways in shaping self-directed learning abilities among undergraduate nursing students

**DOI:** 10.3389/fpsyg.2024.1450462

**Published:** 2024-11-14

**Authors:** Xiangxiang Li, Meifang Wang, Xiujuan Feng, Xiumin Yin, Juan Liang

**Affiliations:** ^1^Department of Nursing and Rehabilitation, Xi'an Jiaotong University City College, Xi'An, China; ^2^Department of Thoracic Surgery, Shandong Provincial Hospital Affiliated to Shandong First Medical University, Jinan, China; ^3^Department of Pediatrics, The First Affiliated Hospital of Air Force Military Medical University, Xi'An, China

**Keywords:** metacognitive ability, psychological capital, interpersonal communication, self-directed learning, nursing student

## Abstract

**Background:**

Developing self-directed learning in undergraduate nursing students affects not only their learning and their lives, but also their future professional development and the quality of their future practice in clinical nursing. Hence, it is paramount to prioritize and cultivate self-directed learning capabilities among undergraduate nursing students, as this not only enhances their academic pursuits but also equips them with essential lifelong learning skills crucial for the dynamic healthcare landscape.

**Objective:**

To delve into the intricate relationship between metacognitive abilities and self-directed learning practices among nursing students, while concurrently examining the mediating roles of psychological capital and interpersonal communication in this interconnected framework. This exploration aims to provide insights into how these factors interplay to influence the self-directed learning capabilities of nursing students.

**Methods:**

A total of 662 undergraduate nursing students from one university in China were selected as participants in the survey, utilizing stratified random sampling between September 2023 and December 2023. Of these, an impressive 639 students (96.52%) provided valid responses. The Metacognitive Assessment Inventory, Adolescent Sense of Psychological Capital Scale, Supportive Communication Scale, and Self-Directed Learning Ability Scale were employed to comprehensively assess the metacognitive abilities, psychological capital, interpersonal communication skills, and self-directed learning capacities of nursing students. Pearson correlation analysis was subsequently utilized to delve into the related relationships among these variables. To test the mediating effects, the Bootstrap method, specifically Model 6 of the SPSS-Process package devised by Hayes, was applied.

**Results:**

The findings revealed a robust positive correlation among metacognitive ability, psychological capital, interpersonal communication ability, and self-directed learning ability, with all associations reaching statistical significance at *P* < 0.01. Notably, the mediating roles of psychological capital and interpersonal communication in the relationship between metacognitive ability and self-directed learning were significant, as evidenced by the analysis (R^2^ = 0.347, F = 67.278, *P* < 0.001). Furthermore, the results indicated that metacognitive ability exerts an indirect influence on self-directed learning through a sequential chain of mediation involving psychological capital and interpersonal communication ability.

**Conclusions:**

This finding highlights the intricate interplay between these factors, suggesting that enhancing nursing students' metacognitive abilities may indirectly bolster their self-directed learning by first fortifying their psychological capital and then fostering stronger interpersonal communication skills.

## 1 Introduction

The World Health Organization (2020), underscored a dire global shortage of 5.9 million nurses, vastly inadequate to meet the burgeoning demand for health services worldwide. Consequently, it urged nations to embark on initiatives aimed at nurturing a larger pool of highly skilled caregivers. Various countries have tailored nurse education and training programs to their unique national contexts (Simes et al., 2024; King et al., 2022; Ma et al., 2024), encompassing strategies like bolstering nursing educational capacity, emphasizing the cultivation of nursing students' specialized knowledge and practical competencies, and offering a comprehensive and diversified curriculum to equip them with a broad professional skill set. Nursing students are under greater academic pressure due to the heavy training tasks, the specificity of service objects, evidence-based practices, and the constant updating of knowledge (Yuhuan et al., 2022; Wan et al., 2023; Yildirim et al., 2017). Studies (Hołda et al., 2019; Custers, 2010) have shown that health care professionals' knowledge declines over time, and this loss of knowledge can have a detrimental effect on patients, so it is critical that education promotes the development of skills associated with lifelong learning (Taylor et al., 2023). Self-directed learning (SDL) stands as the cornerstone for cultivating lifelong learning skills, empowering individuals to take charge of their own educational journey and continuously adapt to the ever-evolving landscape of knowledge and skills (Sb, 2001; Taylor et al., 2023). Developing SDL in undergraduate nursing students affects not only their learning and their lives, but also their future career development and the quality of their future clinical nursing work (Al-Noumani et al., 2023; Dinse et al., 2017). Therefore, it is important to focus on and foster SDL in undergraduate nurses.

## 2 Background

Bindon (2017) asserts that the concept of lifelong learning serves as the fundamental pillar underpinning nursing practice, emphasizing the imperative for nurses to continually enhance their knowledge and skills throughout their careers. Currently, lifelong learning is recognized as an important component of the nursing humanities, but there are still barriers to implementing lifelong learning in nursing practice (Li R. et al., 2023). One of the major barriers is the lack of guidance for nursing students to develop effective learning strategies (Zheng et al., 2020; Zimmerman and Schunk, 2011; Lee et al., 2018). The cultivation of SDL capabilities is a key factor in improving learning strategies for nursing students, enhancing learning outcomes, and facilitating the formation of lifelong learning concepts (Sb, 2001; Millanzi et al., 2021; Li R. et al., 2023). Nursing professionals are mandated to continuously refine their technical acumen and stay abreast of the latest advancements within their discipline, ensuring they deliver the most up-to-date and effective care to patients. Therefore, they need to become lifelong learners and greatly promote the development of their SDL (Alegría et al., 2014; Jin and Ji, 2021). Defined by Knowles in 1975, SDL refers to “the process by which an individual proactively identifies his or her learning needs, sets goals, determines the human and material resources required for learning, selects and implements appropriate learning strategies, and evaluates learning outcomes, with or without the help of others.” In SDL, the process is dynamic, circular, proactive and constructive, and the responsibility for learning shifts to the students, who take an active role in formulating and initiating the actions necessary to achieve their learning goals (Thiede and Dunlosky, 1999; Zimmerman, 2000). Researches (Tekkol and Demirel, 2018; Knowles, 1975) have found that learners with high SDL ability are more conscious of their deficiencies and how to address them. Healthcare professionals with strong SDL capabilities can continue to grow and enhance their knowledge to improve patient care and well-being (Taylor et al., 2023; Tekkol and Demirel, 2018). In addition, Studies have shown that general demographic factors such as gender, educational system, teaching methods, major, performance ranking, family monthly income (Berkhout et al., 2015; Strage, 1998; Li L. et al., 2023) and factors such as self-efficacy, autonomy, critical thinking, academic performance, learning motivation, self-awareness, and metacognition significantly influence SDL (Zhang et al., 2023; Anwar and Muti'ah, 2022; Sukkamart et al., 2023; Hwang and Oh, 2021; Pei et al., 2023; Yeo and Jang, 2023).

Factors affecting SDL include metacognition, which is an important variable. The theory of self-directed learning defines learning as a process guided by metacognition (Zimmerman, 1998; Panadero, 2017). This theory proposes that individuals not only control their cognitive and emotional mechanisms during learning, but also monitor and evaluate their external behavior, adjusting their learning behavior through metacognition (Zimmerman, 1998). Flavell (1979) described metacognition as an individual's awareness of their own cognitive activities and the conscious monitoring, coordination, and integration of their cognitive activities. O'Neil and Abedi (1996) further improved the definition of metacognition by adding the examination and adjustment of learning progress to meet learning goals, and pointed out that metacognition includes four components: planning learning activities, monitoring learning progress, perceiving learning activities, and regulating cognitive strategies to adapt to the learning activities. Previous studies have also confirmed the correlation between metacognition and SDL. A cross-sectional study of 216 Chinese nursing students showed that their self-directed learning ability and metacognitive ability were at a moderate level, and the two were significantly positively correlated (Chen et al., 2019). Another study of 3,047 Chinese five-year vocational nursing students showed that individuals with high metacognitive scores exhibited higher self-directed learning ability (Jin and Ji, 2021). Research by scholars abroad also supports this view (Ewell et al., 2023; Gonullu and Artar, 2014; Ha et al., 2023). While prior studies have ventured into examining the correlation between metacognition and self-directed learning prowess, a significant void remains in unraveling the intricate pathways that underpin this relationship. This research endeavor seeks to delve deeper into the intricate interplay and pathways linking metacognition to self-directed learning among Chinese nursing students. The ultimate goal is to contribute fresh perspectives that can guide the creation of effective methodologies for fostering stronger self-directed learning abilities among these aspiring healthcare professionals.

The definition of psychological capital is a concept proposed by Luthans et al. (2007), within the framework of positive psychology and positive organizational behavior, focusing on the core concept of positive psychological strengths in individuals. They posit that psychological capital encapsulates an individual's positive psychological stance, comprising key facets such as self-confidence or self-efficacy, hope, optimism, and resilience—all of which contribute to a robust mental framework. Metacognition theory suggests that metacognition is an individual's awareness and control of cognitive domains, and as an internal cognitive process, psychological capital is influenced by metacognitive abilities (O'Neil and Abedi, 1996; Spada et al., 2007; Gulistan Yunlu and Clapp-Smith, 2014). Researchers have found a significant positive correlation between metacognitive abilities and individuals' positive psychological emotions (Lin et al., 2023; Bacadini França et al., 2023), and metacognitive abilities can predict the level of psychological capital (Gulistan Yunlu and Clapp-Smith, 2014). In other words, individuals with stronger abilities in planning, monitoring, controlling, and evaluating cognitive activities tend to have richer internal positive psychological emotions and higher levels of psychological capital. The Main Effect Model of psychological capital suggests that it directly influences individuals' attitudes and behaviors, and this has been supported by numerous empirical studies (Luthans and Youssef, 2007). Relevant research has shown that psychological capital can have positive impacts on students' self-directed learning, academic performance, and work performance (Luthans and Jensen, 2012; Hassan et al., 2023; Li R. et al., 2023). Hence, enhancing the level of psychological capital among nursing students has the potential to mitigate negative emotions stemming from academic pressures, amplify positive learning sentiments, intensify the enjoyment derived from learning, and bolster their self-directed learning capabilities. On this premise, the present study formulates the following hypothesis: psychological capital serves as a mediating factor in the relationship between metacognitive abilities and self-directed learning capacity among undergraduate nursing students.

Interpersonal communication ability constitutes a pivotal prerequisite for nurses in the clinical setting, enabling them to effectively engage with patients and colleagues alike. Good interpersonal skills help foster nurse-patient relationships, increase patient confidence in nurses, and improve nursing satisfaction (Höglander et al., 2023). The rise of metacognitive awareness has led to improved interpersonal communication skills (Vuillaume et al., 2020). According to the theory of metacognition, individuals are able to give full play to their metacognition skills in communication, to feel each other's emotions, and to improve their ability to communicate with others (Mason et al., 2022). In addition, related studies have found that improved interpersonal communication promotes self-directed learning, suggesting that interpersonal communication has a positive effect on self-directed learning (Chang and Lai, 2022; Cheng et al., 2010). Thus, interpersonal skills may act as mediators between metacognitive and self-directed learning capabilities.

Concurrently, research underscores a positive correlation between psychological capital and interpersonal communication proficiency, suggesting that individuals with robust mental resources tend to excel in their interpersonal interactions (Liu X. et al., 2023). Individuals with low psychological capital tend to be poor communicators, while individuals with high psychological capital are good at communicating with others and are more confident in their communication, resulting in better relationships (Pelletier Brochu et al., 2018; Liu Y. et al., 2023). According to the theory of the main effect model of psychological capital (Luthans and Youssef, 2007), individuals with high psychological capital often possess higher confidence, hope, optimism, and resilience when faced with setbacks or challenges. They are more likely to leverage their positive psychological advantages, engage in communication with others, seek positive resources or assistance, and demonstrate stronger interpersonal skills. Drawing upon the aforementioned speculations, it is plausible that psychological capital and interpersonal communication ability may exert a sequential mediating influence, forming a chain that connects metacognition to self-directed learning abilities.

Guided by the preceding analysis, this study endeavors to unravel the intricate relationships among metacognition, psychological capital, interpersonal communication ability, and self-directed learning abilities among nursing students. Our objective is to illuminate the underlying paths through which metacognition influences the self-directed learning capacity of nursing students. In line with existing research, we posit the following hypotheses:

1. Metacognition is positively associated with self-directed learning ability among nursing students.
2. Psychological capital functions as a mediating variable in the relationship between metacognition and self-directed learning ability.
3. Interpersonal communication ability also serves as a mediating factor linking metacognition to self-directed learning ability.
4. The influence of metacognition on nursing students' self-directed learning abilities is mediated sequentially by psychological capital and interpersonal communication ability, forming a chain mediation effect.

## 3 Methods

### 3.1 Subjects

Between September and December 2023, undergraduate nursing students enrolled in a university located in Shaanxi Province, China, were selected as the target population for this survey. A stratified random sampling approach was employed, with stratification based on academic year (freshman, sophomore, junior, and senior). The inclusion criteria stipulated that participants must be: (1) four-year undergraduate nursing students and (2) willing to provide informed consent voluntarily. Conversely, exclusion criteria were applied to exclude students who were not currently enrolled due to reasons such as suspension from school or military service.

To determine the appropriate sample size for this study, the formula N = Z ^2^ × [P × (1-P)]/E ^2^ was utilized, where N represents the sample size, Z is the statistic, E is the margin of error, and P is the estimated probability. With Z set at 1.96 to ensure a 95% confidence level and E at 4% for a precise estimation. Drawing from prior research, the prevalence rate of self-directed learning ability among medical students has been reported to be 54.3% (Juan et al., 2023). Incorporating a 10% attrition rate to account for potential dropouts, the calculated sample size required for this study was 662 participants.

### 3.2 Survey tool

#### 3.2.1 Metacognitive assessment inventory (MAI)

Metacognitive Assessment Inventory (MAI) put forward by Schraw and Dennison (1994) and revised to Chinese by Guo (2009) and reported the Cronbach's α 0.953, was utilized to evaluate the individual's metacognitive level. The scale encompasses two key dimensions: cognitive knowledge and cognitive management, comprising a comprehensive total of 52 items. Employing the Likert 5-point scale, where 1 signifies “never” and 5 denotes “always,” participants rate their responses, resulting in a total score range of 52 to 260. Notably, a higher score on this scale indicates a more pronounced level of metacognitive awareness. In the context of this study, the reliability of the scale was validated through a Cronbach's α coefficient of 0.973, indicating a high degree of internal consistency and reliability.

#### 3.2.2 The positive psychological capital questionnaire (PPQ)

The Positive Psychological Capital Questionnaire (PPQ) compiled by Zhang et al. (2010) was utilized to measure students' psychological capital. The questionnaire comprehensively assesses four dimensions of psychological capital: self-efficacy (7 items), hope (6 items), optimism (6 items), and resilience (7 items), totaling 26 items in all. Utilizing a Likert 6-point scale ranging from “completely inconsistent” (1 point) to “completely consistent” (6 points), participants' responses are scored, resulting in a total score that spans from 26 to 156. This scoring system indicates that a higher total score corresponds to a greater level of psychological capital among the subjects. Drawing upon previous research (Cao and Li, 2019), this scale has demonstrated robust reliability and validity. In the present study, the Cronbach's α coefficient for the scale was 0.926, further attesting to its internal consistency and reliability.

#### 3.2.3 The supportive communication scale (SCS)

The Supportive Communication Scale (SCS) developed by Whetten and Cameron (1998) and translated to Chinese version by Yuan (2008), was used to measure interpersonal communication ability. In previous study it was reported good Cronbach's α coefficient with 0.879 (Zhao et al., 2019). The scale encompassed three essential dimensions: counseling and consultation, administering effective negative feedback, and fostering supportive communication, consisting of a comprehensive 20 items. Adhering to a Likert 5-point scale, where 1 represents “completely disagree” and 5 signifies “completely agree,” participants' responses were scored, yielding a total score range of 20 to 100. Notably, a higher score on this scale is indicative of a stronger interpersonal communication ability among the subjects. In the context of this study, the reliability of the scale was confirmed through a Cronbach's α coefficient of 0.889, signifying good internal consistency and reliability.

#### 3.2.4 Self-directed learning ability scale (SLAS)

Self-directed Learning Ability Scale (SLAS) designed by Lin and Jiang (2004) and reported the Cronbach's α coefficient was 0.860, was applied to assess the self-directed learning ability of nursing students. The scale comprehensively assesses three pivotal dimensions of learner autonomy: self-management ability, information literacy, and learning and cooperative abilities, encompassing a total of 28 items. Utilizing a Likert 5-point scale, where 1 signifies “complete non-conformity” and 5 represents “complete conformity,” participants' responses are scored. The resulting total score serves as an indicator of the learner's self-directed learning ability, with higher scores reflecting a greater level of autonomy. Notably, the scale has demonstrated reliability in this study, with a Cronbach's α coefficient of 0.822, underscoring its internal consistency and validity.

### 3.3 Survey methods

1) The sample size was determined relative to the overall population size, resulting in a ratio of 662:1,192, which can be simplified to 331:596 for clarity. This ratio guided the selection process.
2) Based on this sample ratio, we systematically identified the number of freshmen, sophomores, juniors, and seniors to be included in the study. The respective numbers were 72, 48, 281, and 261, ensuring a representative distribution across academic years.
3) To ensure randomness in sampling, we employed a simple random sampling technique within each grade level. Specifically, students who met the inclusion criteria within a given grade were numbered sequentially. These numbers were then written on number tags and placed in a non-transparent container, which was thoroughly mixed. Subsequently, without replacement, we sequentially drew the required number of tags (m) from the container, corresponding to the pre-determined sampling ratio. The students associated with these tags were selected as the survey participants for that particular grade. This process was repeated for all grades to obtain the desired sample.

After undergoing comprehensive training, the investigators meticulously explained the purpose and content of the study to school administrators, seeking their consent. During the autumn semester of 2023 (specifically between September and December), dedicated class time was allocated to provide a unified and clear explanation of the survey's objectives. Students were reassured that their responses would be used solely for research purposes and that their personal information would be kept strictly confidential. Once informed consent was obtained, students were guided to begin completing the questionnaire. The questionnaire was initially distributed to undergraduate students via the Questionnaire Star platform, allowing for efficient data collection and rigorous quality control by the investigators. Ultimately, a total of 662 questionnaires were administered, resulting in 639 valid responses, yielding an impressive effective recovery rate of 96.52%.

### 3.4 Ethics statement

The present study was approved by the Ethics Committee of Tangdu Hospital (No.: TDLL-202210-17). The attributes, benefits, uses, and disadvantageous effects of the study were explained to all participants and informed consent was also obtained.

### 3.5 Data processing

Data manipulation was conducted using SPSS 26.0 software. The Kolmogorov-Smirnov (K-S) test and the Shapiro-Wilk (S-W) test were conducted to assess the normality of the data distribution for metacognition, psychological capital, interpersonal communication skills, and self-directed learning abilities, revealing *p* > 0.05, which indicates that the data adhere to a normal distribution. Counting data was presented in a clear and concise manner, utilizing frequency and percentage for easy interpretation. For measuring data, mean values accompanied by standard deviations were reported, providing a comprehensive understanding of the distribution and variability. To compare the scores of nursing undergraduates across various demographic categories, either the *t*-test or ANOVA was selected based on the nature of the data, ensuring accurate and meaningful comparisons. Furthermore, to explore the intricate relationships among metacognition, psychological capital, interpersonal communication ability, and self-directed learning ability, Pearson correlation analysis was conducted. To delve deeper into the potential mediating effects, the Bootstrap method was utilized in conjunction with Model 6 of the SPSS-Process macro developed by Hayes (2013). This advanced approach facilitated the rigorous testing and analysis of mediating relationships, contributing to a more comprehensive understanding of the underlying mechanisms. All statistical analyses were conducted with a significance level set at *P* < 0.05, ensuring that any observed differences or associations were statistically meaningful and could be confidently interpreted.

## 4 Results

### 4.1 Common method bias assessment

To address the potential issue of common method bias in this study, the Harman single-factor test was utilized as a rigorous assessment tool. From the 100 items under consideration, 17 factors with characteristic values exceeding 1 were extracted, with these factors indicating statistical significance. Notably, the percentage of variance explained by the first common factor, which often serves as an indicator of common method bias, was found to be 23.94%. This value, being less than the critical threshold of 40%, was considered as evidence that the influence of common method variance in this study is not substantial and, therefore, not likely to significantly confound the results. Based on this analysis, it can be confidently concluded that these findings are not critically threatened by common method bias.

### 4.2 Comparison of scores across demographic characteristics in nursing undergraduates

The study encompassed a diverse sample of nursing undergraduates, consisting of 26 males and 613 females, spanning across academic years: 70 freshmen, 47 sophomores, 273 juniors, and 249 seniors. A comprehensive analysis was conducted to examine potential differences in various scales, including self-directed learning, metacognition, psychological capital, and interpersonal communication abilities, among these students based on their gender and academic grade.

The results revealed no statistically significant variations (all *P* > 0.05) in any of the aforementioned scales between nursing undergraduates of different genders or academic grades. For a detailed breakdown of the specific scores and comparisons, please refer to Table 1.

**Table 1:** Comparative analysis of scores across demographic characteristics among nursing undergraduates in various scales.

**Variable**		***n***	**Psychological capital**	**Interpersonal communication**	**Metacognition**	**Self-directed learning ability**
Sex	Male	26	98.15 ± 23.45	69.38 ± 11.06	81.00 ± 20.77	89.54 ± 11.90
	Female	613	99.89 ± 15.99	65.90 ± 10.63	80.22 ± 14.82	87.22 ± 9.31
	t		−0.374	1.633	0.190	0.980
	*P*		0.711	0.103	0.851	0.336
Grade	Freshman	70	99.83 ± 18.36	65.71 ± 10.42	78.86 ± 16.50	87.06 ± 9.53
	Sophomore	47	99.94 ± 18.82	66.21 ± 9.44	76.09 ± 15.72	86.34 ± 10.34
	Junior	273	98.79 ± 15.26	65.72 ± 10.63	81.01 ± 13.26	87.00 ± 8.64
	Senior	249	100.92 ± 16.42	66.47 ± 11.00	80.59 ± 16.34	87.92 ± 10.05
	F		0.737	0.241	1.672	0.627
	*P*		0.530	0.868	0.172	0.598

### 4.3 Correlation analysis

A thorough correlation analysis was conducted to explore the relationships among the key variables under study: metacognition, psychological capital, interpersonal communication ability, and self-directed learning ability. The results of this analysis revealed a positive and significant correlation among all pairs of these variables, indicating that they are intimately linked and mutually reinforcing.

Table 2 presents a comprehensive overview of the descriptive statistics for each variable, including the mean, standard deviation, and correlation coefficients. This table provides a clear and concise visualization of the strength and direction of the relationships between these important constructs, further substantiating the interconnectedness of metacognition, psychological capital, interpersonal communication, and self-directed learning in the context of nursing undergraduate education.

**Table 2:** Correlational analysis of metacognition, psychological capital, interpersonal communication, and self-directed learning ability among nursing undergraduates (*N* = 639).

**Variables (value range)**	**Mean**	**SD**	**1**	**2**	**3**	**4**
1. Metacognition (24, 120)	80.25	15.09	1			
2. Psychological capital (51, 154)	99.82	16.34	0.492^**^	1		
3. Interpersonal communication ability (25, 98)	66.05	10.66	0.324^**^	0.484^**^	1	
4. Self-directed learning ability (54, 125)	87.32	9.43	0.440^**^	0.481^**^	0.468^**^	1

^**^P <0.01, SD means standard deviation.

### 4.4 Mediation effect test

The mediation effects of psychological capital and interpersonal communication ability on the relationship between metacognition and self-directed learning ability among nursing students were examined, as presented in Table 3. To ensure the validity of the findings, the analysis was conducted using the SPSS macro program Process, developed by Hayes, while controlling for potential confounding variables such as gender and academic grade.

**Table 3:** Regression analysis of relationships between key variables.

**Regression equation**		***R***	***R ^2^***	***F***	**Significance of regression coefficient**	
**Outcome variable**	**Predictive variable**				β	***t***
Psychological capital		0.493	0.243	67.845^***^		
	Metacognition				0.533	14.235^***^
Interpersonal communication ability		0.500	0.250	52.732^***^		
	Metacognition				0.078	2.783^**^
	Psychological capital				0.281	10.912^***^
Self-directed learning ability		0.589	0.347	67.278^***^		
	Metacognition				0.147	6.347^***^
	Psychological capital				0.133	5.722^***^
	Interpersonal communication ability				0.246	7.489^***^

^**^P <0.01, ^***^P <0.001.

The regression analysis yielded several insightful results. Firstly, metacognition was found to have a direct and statistically significant positive predictive effect on both psychological capital (β = 0.533, *P* < 0.001) and interpersonal communication ability (β = 0.078, *P* < 0.01). Furthermore, psychological capital emerged as a direct and positive predictor of interpersonal communication ability (β = 0.281, *P* < 0.001).

Additionally, the analysis revealed that metacognition exerts a direct and positive influence on self-directed learning ability (β = 0.147, *P* < 0.001). Similarly, both psychological capital (β = 0.133, *P* < 0.001) and interpersonal communication ability (β = 0.246, *P* < 0.001) were identified as direct and positive predictors of self-directed learning ability.

These findings collectively suggest a complex interplay between metacognition, psychological capital, interpersonal communication ability, and self-directed learning ability, with the former variables mediating and reinforcing the latter.

As depicted in Table 4, the mediation model was rigorously evaluated adhering to the methodology outlined by Hayes. To fortify the assessment of the mediation effects, the 95% confidence interval was meticulously computed through a Bootstrap resampling technique, replicated 5,000 times. The findings unequivocally demonstrated the notable mediation roles of psychological capital and interpersonal communication ability, with a mediation effect size of 0.127.

**Table 4:** The mediating role of psychological capital & interpersonal skills in boosting self-directed learning through metacognition.

	**Value**	**Boot standard error**	**95% CI (lower limit)**	**95% CI** **(upper limit)**	**Relative mediation effect**
Total	0.127	0.020	0.087	0.168	46.35%
Ind 1: X → M1 → Y	0.071	0.018	0.037	0.107	25.91%
Ind 2: X → M2 → Y	0.019	0.009	0.002	0.036	6.93%
Ind 3: X → M1 → M2 → Y	0.037	0.010	0.020	0.057	13.50%
Ind 1- Ind 2	0.052	0.020	0.016	0.091	
Ind 1- Ind 3	0.034	0.022	−0.009	0.078	
Ind 2- Ind 3	−0.018	0.013	−0.047	0.003	

N = 639. Number of bootstrap samples for bias corrected bootstrap confidence intervals: 5,000. Level of confidence for all confidence intervals: 95%.

X, metacognition; M1, psychological capital; M2, interpersonal communication ability; Y, Self-learning ability.

This mediation unfolds along three distinct pathways: Firstly, an indirect effect (0.071) traverses from metacognition, fostering psychological capital, which subsequently enhances self-directed learning ability. Notably, the exclusion of zero from the Bootstrap 95% confidence interval underscores the statistical significance of psychological capital's mediating role.

Secondly, another indirect effect (0.019) emanates from metacognition, bolstering interpersonal communication ability, which in turn bolsters self-directed learning ability. Likewise, the non-inclusion of zero within the 95% confidence interval, as determined by the Bootstrap method, underscores the significant mediation by interpersonal communication ability.

Lastly, a third indirect effect (0.037) emerges, weaving a more intricate chain from metacognition through psychological capital, then interpersonal communication ability, ultimately nurturing self-directed learning ability. Again, the absence of zero from the Bootstrap 95% confidence interval underscores the collective and significant role played by both psychological capital and interpersonal communication ability in this sequential mediation process between metacognition and self-directed learning ability.

**Figure 1:**
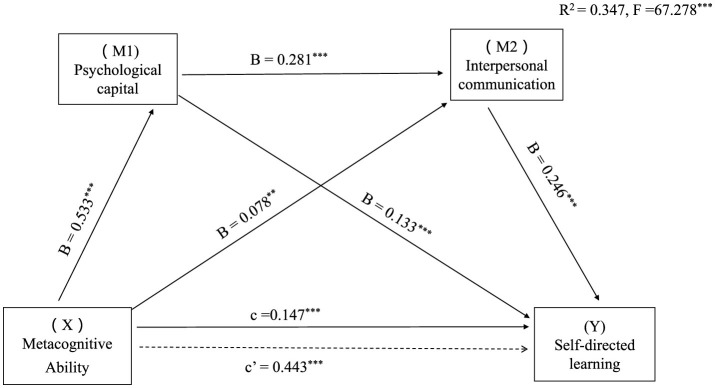
Serial-multiple mediation of psychological capital and interpersonal communication in the relationship between metacognitive ability and self-directed learning with non-standardized beta values. ^**^P < 0.01, ^***^P < 0.001.

Figure 1 visually illustrates the precise trajectory of how nurse students' metacognition influences their self-directed learning ability, underscoring the pivotal roles of psychological capital and interpersonal communication ability in this intricate interplay.

## 5 Discussion

### 5.1 Unraveling the relationship between metacognition and self-directed learning ability

The present study embarked on an exploration to uncover the intricate relationship that exists between metacognition and self-directed learning ability. The findings reveal a striking and significant positive correlation between these two pivotal constructs, thereby providing empirical support for Hypothesis 1 of this research endeavor. This outcome underscores the interdependence and mutually reinforcing nature of metacognition and self-directed learning ability, emphasizing their critical roles in fostering autonomous and effective learning processes. It was consistent with previous research results (Panadero, 2017; Jin and Ji, 2021), that was, the higher the level of metacognitive awareness, the higher the level of self-directed learning ability. Drawing upon the tenets of the humanistic theory, as espoused by Maslow (1943), the process of learning is conceptualized as a holistic endeavor that transcends mere intellectual pursuits. It encompasses a spiritual dimension, underscoring the unity of knowledge and the individual's inner landscape. In this framework, learning and cognition necessitate not just a deliberate examination of factual knowledge but also an intentional investment of emotional energy. Only through the harmonious integration of these two facets—the cognitive and the emotional—can a comprehensive and fulfilling learning experience be achieved, thereby completing the full spectrum of cognitive activity. Some studies have found that metacognition plays an active role in monitoring and regulating the learning process (Ewell et al., 2023; Ha et al., 2023; Chen et al., 2019), which is of great significance to the exploration of other potential qualities of students, it is also a predictor of self-directed learning ability. When confronted with similar learning tasks, individuals endowed with robust metacognitive abilities adeptly leverage their extensive repertoire of skills, which encompass planning, monitoring, and adjustment. These skills manifest across various stages of cognitive endeavors, fostering a steady progression in learning activities and fostering a heightened enthusiasm for autonomous learning among students (Zimmerman, 1998; Flavell, 1979). Conversely, those with weaker metacognitive abilities often lack the capacity for self-monitoring and adjusting their learning strategies, predisposing them to rigid thinking patterns. Consequently, students with lower metacognitive proficiency are more prone to encountering obstacles, which can diminish their interest in self-directed learning (Siqueira et al., 2020; Xiang and Ding, 2013).

In light of these insights, educators can play a pivotal role by integrating metacognitive skills into learning activities. For example, nursing educators can incorporate reflective practice into nursing education, motivating students to regularly examine their learning processes and strategies, and providing personalized feedback and guidance to help them gain insights into their learning strengths and weaknesses, thereby enhancing their metacognitive abilities and fostering self-directed learning. This approach fosters a more dynamic and adaptable learning environment, where students are not only equipped with the knowledge but also the tools to navigate and excel in their academic pursuits.

### 5.2 The singular mediation role of psychological capital and interpersonal communication ability in the relationship between metacognition and self-directed learning ability among nursing students

The present study uncovered a pivotal finding: psychological capital serves as a mediating factor in the nexus between metacognition and self-directed learning ability among nursing students, thereby validating Hypothesis 2 of the research. Metacognitive ability could positively predict psychological capital, which is similar to previous studies (Dapp and Roebers, 2021; Gulistan Yunlu and Clapp-Smith, 2014). This phenomenon suggests that psychological capital, as an intrinsic positive psychological resource, can act through self-regulation of the internal factors of the individual, which provides empirical support for the main effect model of psychological capital (Luthans and Youssef, 2007; Lin et al., 2023; Bacadini França et al., 2023). Furthermore, this study revealed a marked and statistically significant positive correlation between the level of psychological capital possessed by nursing students and their self-directed learning ability. This conclusion indicates that psychological capital can directly affect individual attitudes and behaviors, which is consistent with some previous research results (Luthans and Jensen, 2012; Hassan et al., 2023; Li R. et al., 2023), and has important significance for the cultivation of self-directed learning ability. Additionally, the study's findings imply the necessity of implementing targeted intervention measures aimed at enhancing the psychological capital of nursing students who exhibit lower levels of metacognitive ability. For example, nursing educators can bolster students' psychological capital by creating a supportive, inclusive environment, conducting workshops on stress, time management, and goal setting, and acknowledging students' achievements to enhance nursing students' self-directed learning abilities.

This study further delved into the complexities of learning dynamics among nursing students, uncovering a significant mediating role of interpersonal communication ability in the relationship between metacognition and self-directed learning ability. This finding not only validates Hypothesis 3 but also aligns with recent scholarly discourse, which positions metacognition as a cornerstone of effective interpersonal communication (Vuillaume et al., 2020; Flavell, 1979). Our results echo previous research, demonstrating a positive correlation between metacognitive ability and interpersonal communication skills, emphasizing the interconnectedness of these cognitive processes. Specifically, we found that nursing students with heightened metacognitive levels exhibited greater awareness and proficiency in effective communication strategies. Compared to their peers with lower metacognitive abilities, these students possessed superior interpersonal communication skills, which, in turn, bolstered their self-directed learning abilities. This phenomenon can be attributed to their ability to discern and navigate the diverse cognitive and behavioral responses of individuals when confronted with similar challenges. In the realm of learning and cognition, nursing students adept at interpersonal communication tend to adopt multifaceted perspectives, leveraging their communication skills to gather a wider array of social resources when faced with decision-making situations. This approach facilitates smoother cognitive processes and significantly heightens their interest in self-directed learning (Liu Y. et al., 2023; Chang and Lai, 2022; Cheng et al., 2010). Nursing educators can enhance students' interpersonal skills by incorporating role-playing, group discussions, empathy training, active listening, and encouraging extracurricular activities like volunteering, fostering effective communication and patient-centered care to improve nursing students' self-directed learning abilities. Our findings underscore the importance of fostering not only metacognition but also interpersonal communication abilities among nursing students, as both contribute to the development of robust self-directed learning capabilities, ultimately enhancing their academic and professional success.

### 5.3 The sequential mediation of psychological capital and interpersonal communication ability in the relationship between metacognition and self-directed learning ability among nursing students

This study contributes to the existing literature by uncovering a sequential mediation effect of psychological capital and interpersonal communication ability in the intricate relationship between metacognition and self-directed learning ability among nursing students. This finding serves as a validation of Hypothesis 4, extending the understanding of the interconnectedness among these variables. Prior research (Zhang et al., 2023; Pei et al., 2023; Yeo and Jang, 2023; Siqueira et al., 2020; Gulistan Yunlu and Clapp-Smith, 2014; Vuillaume et al., 2020) has established the correlations among metacognition, psychological capital, interpersonal communication ability, and self-directed learning ability, yet few studies have delved into the intricate mechanisms underlying these relationships.

Our study builds upon this foundation, offering a novel perspective by elucidating the sequential mediation pathway. Specifically, we found that nursing students' metacognition exerts an influence on their self-directed learning ability, with psychological capital and interpersonal communication ability functioning as sequential mediators. This finding highlights the importance of recognizing that education, particularly in the context of “exam-oriented” systems, often prioritizes learning outcomes (i.e., self-directed learning ability) while overlooking the critical role of individual's active evaluation, adjustment, and feedback mechanisms (including metacognition, psychological capital, and interpersonal communication ability) in the learning process, strategy application, effect monitoring, resource acquisition, and learning collaboration. This oversight could contribute to the prevalent issue of “high scores but low abilities” (Long, 2016).

When nursing students possess strong metacognitive abilities, they demonstrate a heightened propensity to harness both internal and external positive resources within their cognitive endeavors, actively seeking and integrating these resources to enhance their learning experiences (Knowles, 1975; O'Neil and Abedi, 1996). Consequently, they exhibit elevated levels of self-efficacy, resilience, hope, and optimism—collectively known as individual positive psychological qualities—which signify a heightened state of psychological capital. This abundant psychological capital, in turn, serves as a catalyst, motivating students to actively seek out internal and external resources that facilitate the establishment and nurturing of interpersonal relationships, thereby refining their interpersonal communication skills (O'Neil and Abedi, 1996; Spada et al., 2007).

Essentially, a strong psychological capital foundation empowers nursing students to excel in interpersonal relationships and communication, allowing them to tap into a wider range of social resources. With their proficient communication skills, they can effectively navigate the learning environment, improving the effectiveness of their self-directed learning and ultimately cultivating a more productive and rewarding educational experience. In the context of the ongoing global nurse shortage and its potential impact on new nurses contemplating leaving the profession, it is imperative to acknowledge that nurses equipped with robust metacognition, psychological capital, and interpersonal communication skills are more resilient, adaptable, and job-satisfied. These attributes can aid them in managing the challenges and pressures of nursing, thereby mitigating the risk of burnout and turnover.

## 6 Limitations

This study, while insightful, acknowledges several limitations that are crucial for future research endeavors. Firstly, the intricate interplay between metacognition and self-directed learning ability is multifaceted, yet the study solely delves into the mediating role of psychological capital and interpersonal communication abilities. This narrow focus fails to comprehensively unravel the intricate model of influencing factors that shape self-directed learning ability. Hence, future research should incorporate a broader array of relevant variables to construct a more comprehensive and nuanced model that captures the full extent of this relationship.

Secondly, being a cross-sectional study, the investigation is constrained to examining the relationships between variables at a single point in time. This approach limits our ability to trace the dynamic evolution of these relationships and establish clear causal links. Longitudinal studies, which track changes over extended periods, are thus imperative to unravel the intricate mechanisms at play between these variables and provide a more robust understanding of their temporal dynamics.

Lastly, the sample used in this study comprises exclusively undergraduate nursing students from one university, potentially limiting the generalizability of our findings. To enhance the universality of the conclusions, a multicenter, multi-regional study encompassing a wider array of undergraduate nursing students is highly recommended. Such an approach would bolster the representativeness of our sample and ensure that the insights gained are applicable to a broader range of nursing education contexts.

## 7 Conclusion

This study meticulously examines the intricate relationship between metacognition and self-directed learning ability among nursing students, offering valuable insights into their educational development. Firstly, these findings unequivocally demonstrate that metacognition serves as a significant predictor of self-directed learning ability, emphasizing its pivotal role in fostering autonomous learning.

Secondly, an exploration into the underlying mechanisms uncovers the mediation effects of psychological capital and interpersonal communication ability within this relationship. The findings indicate that both psychological capital and interpersonal communication ability independently serve as pivotal mediators, bridging the divide between metacognition and self-directed learning ability. This highlights the significance of fostering these elements to augment students' capacity for self-directed learning.

Furthermore, the analysis uncovers a profound chain mediating effect, where psychological capital and interpersonal communication ability sequentially influence the relationship between metacognition and self-directed learning ability. This finding highlights the interconnectedness of these variables and underscores the need for a holistic approach in fostering nursing students' learning autonomy.

In accordance with these findings, it is recommended that nursing educators prioritize the incorporation of mediating variables into their instructional strategies. Specifically, they ought to contemplate executing targeted interventions designed to strengthen students' psychological capital and elevate their interpersonal communication abilities. Through this approach, educators can indirectly foster the development of self-directed learning ability, particularly in situations where directly nurturing metacognition may pose challenges.

## Data Availability

The raw data supporting the conclusions of this article will be made available by the authors, without undue reservation.
